# Congenital Anomalies Programmed by Maternal Diabetes and Obesity on Offspring of Rats

**DOI:** 10.3389/fphys.2021.701767

**Published:** 2021-08-10

**Authors:** Vanessa Caruline Araujo-Silva, Alice Santos-Silva, Andressa Silva Lourenço, Cristielly Maria Barros-Barbosa, Rafaianne Queiroz Moraes-Souza, Thaigra Sousa Soares, Barshana Karki, Verônyca Gonçalves Paula, Yuri Karen Sinzato, Débora Cristina Damasceno, Gustavo Tadeu Volpato

**Affiliations:** ^1^Laboratory of System Physiology and Reproductive Toxicology, Institute of Biological and Health Sciences, Federal University of Mato Grosso, Barra do Garças, Brazil; ^2^Laboratory of Experimental Research on Gynecology and Obstetrics, Postgraduate Program on Tocogynecology, São Paulo State University, Botucatu, Brazil

**Keywords:** hyperglycemia, obesity, pregnancy, biochemical, malformation, rat

## Abstract

Embryo-fetal exposure to maternal disorders during intrauterine life programs long-term consequences for the health and illness of offspring. In this study, we evaluated whether mild diabetic rats that were given high-fat/high-sugar (HF/HS) diet presented maternal and fetal changes at term pregnancy. Female rats received citrate buffer (non-diabetic-ND) or streptozotocin (diabetic-D) after birth. According to the oral glucose tolerance test (OGTT), the experimental groups (*n* = 11 animals/group) were composed of non-diabetic and diabetic receiving standard diet (S) or HF/HS diet. High-fat/high-sugar diet (30% kcal of lard) in chow and water containing 5% sucrose and given 1 month before mating and during pregnancy. During and at the end of pregnancy, obesity and diabetes features were determined. After laparotomy, blood samples, periovarian fat, and uterine content were collected. The diabetic rats presented a higher glycemia and percentage of embryonic losses when compared with the NDS group. Rats DHF/HS presented increased obesogenic index, caloric intake, and periovarian fat weight and reduced gravid uterus weight in relation to the other groups. Besides, this association might lead to the inflammatory process, confirmed by leukocytosis. Obese rats (NDHF/HS and DHF/HS) showed higher triglyceride levels and their offspring with lower fetal weight and ossification sites, indicating intrauterine growth restriction. This finding may contribute to vascular alterations related to long-term hypertensive disorders in adult offspring. The fetuses from diabetic dams showed higher percentages of skeletal abnormalities, and DHF/HS dams still had a higher rate of anomalous fetuses. Thus, maternal diabetes and/or obesity induces maternal metabolic disorders that contribute to affect fetal development and growth.

## Introduction

*Diabetes mellitus* (DM) is a syndrome that is a growing health problem, accounting for 10.4% of global mortality. In 2015, hyperglycemia during pregnancy was observed in 16.2% of women (Cho et al., 2018). In the first few weeks of pregnancy, maternal diabetes is intensely linked to higher number of spontaneous abortions and major congenital malformations (Kitzmiller et al., 1996; Ray et al., 2001).

Fetal programming is a theory that suggests that the environment around the developing fetus plays an important role in determining the risk of disease in childhood and adulthood (Entringer et al., 2012). In this sense, factors such as overweight, obesity, and maternal diabetes during pregnancy are known to be effective agents leading to chronic-disease development in offspring (Yessoufou and Moutairou, 2011), showing the relevance of intrauterine environment for health of descendants in the future.

To reproduce maternal hyperglycemia found in Type 2 DM in animal models, streptozotocin (STZ) induction can be performed in the neonatal period of rats (Tsuji et al., 1988; Jawerbaum and White, 2010; Santos et al., 2015; Bequer et al., 2018; Bueno et al., 2020). This type of experimental diabetes is termed as “mild diabetes” (Hauschildt et al., 2018; Machado et al., 2020). Besides diabetic status, a change in lifestyle, especially in dietary patterns related with growing consumption of industrialized foods (high in calories and fat), affects diabetic progress at long term (Hu, 2011; Ley et al., 2014; Popkin, 2015; Krishan et al., 2018). Animals that are fed high-fat (HF) and/or high-sugar (HS) diets showed metabolic changes, such as increased concentrations of glucose, triglycerides (TG), total cholesterol (TC), and obesity (Matias et al., 2018; Zhao et al., 2019). The offspring of mothers who consumed the HF/HS diet had greater fat tissue, glycemia, TG, and TC levels (Martins Terra et al., 2020). Despite the knowledge about the single effect of HF/HS diet and diabetes on the metabolic response in animals, there are still few studies exploring the association of these two variables during pregnancy. Considering that pregnancy is a critical period, where maternal conditions and habits can lead to persistent changes in offspring (Fleming et al., 2015), it is important that the studies are conducted in a manner where new care strategies can be taken.

Thus, the hypothesis of this study is that diabetic rats submitted to the HF/HS diet before and during pregnancy will present exacerbated damage on a biochemical profile, leading to impaired maternal-fetal relationship. Therefore, the aim of this study was to evaluate maternal and fetal repercussions of the diabetes associated with an HF/HS diet offered before and during pregnancy of rats.

## Materials and Methods

### Animals

Female Wistar rats (230 ± 250 g) were obtained from the Center for Maintenance of Experimental Animals of our Institution, and were maintained under standard laboratory conditions (22 ± 3°C, 12-h light/dark cycle), with pelleted food (Purina rat chow, Purina®, São Paulo State, Brazil) and tap water *ad libitum*. The local Ethical Committee for Animal Research authorized and approved all the procedures and animal handling (Protocol No. 23108.022251/2019-61).

**Figure 1:**
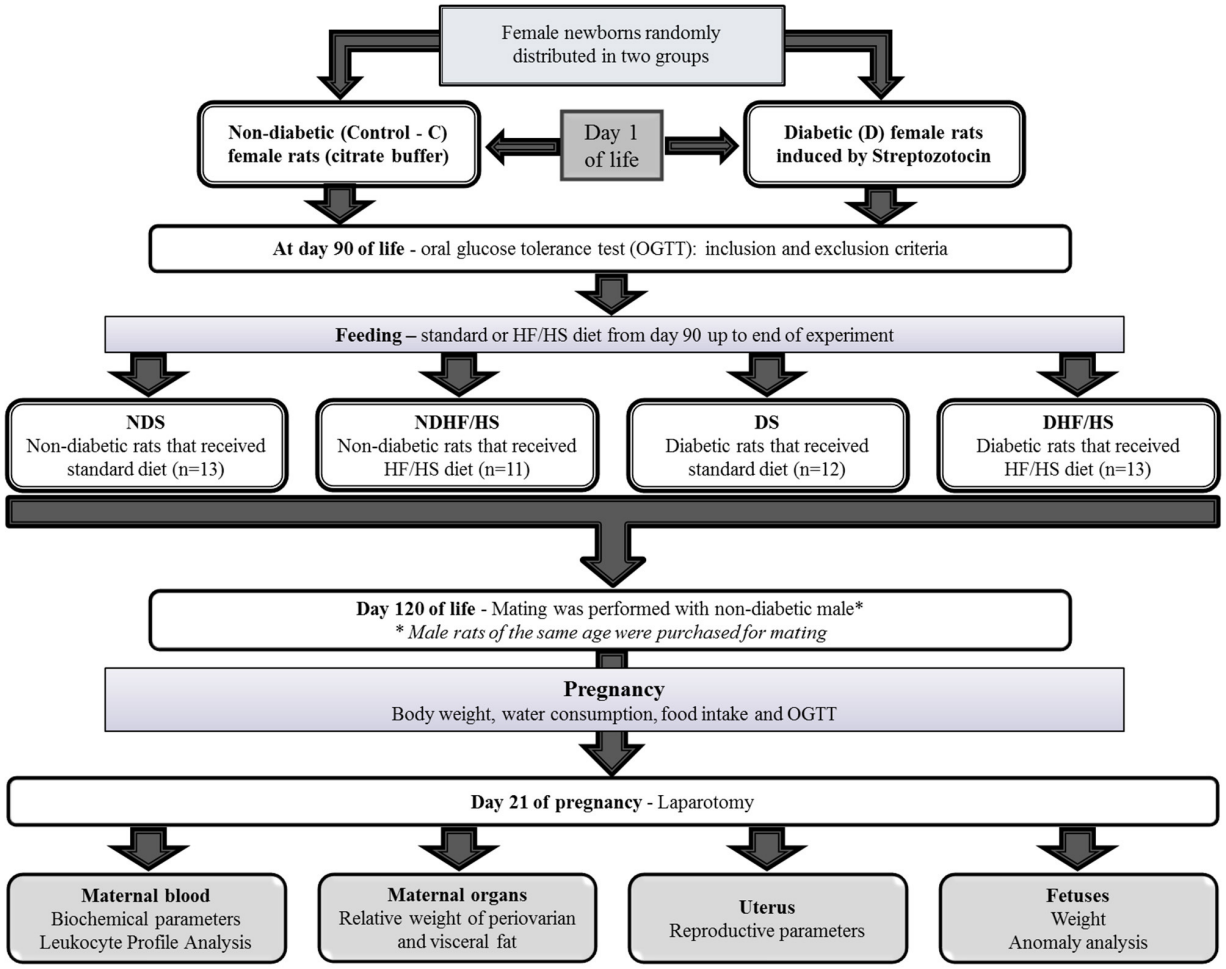
Experimental design.

After 1 week of acclimatization, the females were mated with the male rats with similar age (ratio 3:1) to obtain offspring for induction of diabetes. The experimental sequence of the experiment is summarized in Figure 1.

### Induction and Confirmation of Diabetes

For diabetes (D) induction, half of each female litter was injected with Streptozotocin (100 mg/kg, sc., Sigma–Aldrich, St. Louis, MO, USA) diluted in citrate buffer (.1 mol/L, pH 4.5) on the first day of life (24 h after delivery) (Soares et al., 2021) to induce beta cell necrosis, reproducing glycemic levels similar to Type 2 DM. Non-diabetic (ND) animals were injected with only citrate buffer to simulate the conditions of the STZ-induced group. At day 90 of life, oral glucose tolerance test (OGTT) was performed for inclusion or exclusion of rats to ND and D groups. This test is a marker routinely used in the clinic for diagnosing diabetic status. Then, the rats were fasted for 6 h; after which, a drop of blood was collected from the tail of the rats to determine glycemia (time 0), using a conventional glucometer. The rats were intragastrically given glucose solution (.2 g/m) at a dose of 2-g/kg body weight and after 30, 60, and 120 min later, the blood glucose levels were determined (Neto et al., 2020).

In order to determine the inclusion and exclusion criteria, the standards established by Gallego et al. (2018) were used, and modified from diabetes classification parameters suggested by the American Diabetes Association (2020). For the control group, only the rats with glycemia <140 mg/dL in different time points during OGTT were included. For the diabetic group, the rats presenting least one-time point with glycemia ≥ 200 mg/dL after overload glucose during OGTT were included in the group. The female animals that did not present OGTT with these characteristics for inclusion in the control or diabetic group were excluded and euthanized.

### Experimental Groups

Considering the four experimental groups, and based on previous experiments conducted in our laboratory in relation to reproductive parameters, using 90% power and error type I of 5%, the effect size was determined. Based on the effect size, the sample size was 11 rats per group.

After inclusion and exclusion criteria, the rats (90 days of life = adulthood) were randomized in the experimental groups: non-diabetic rats that received standard diet (NDS); non-diabetic rats receiving high-fat/high-sugar diet (NDHF/HS); diabetic rats, given standard diet (DS); and diabetic rats that received high-fat/high-sugar (HF/HS) diet.

### Standard or High-Fat/High-Sugar Diet

Females from non-diabetic and diabetic dams randomly received standard diet (commercial food: 28.54% Kcal of protein, 62.65% Kcal of carbohydrate, 8.7% Kcal of fat (Purina rat chow, Purina®, Brazil) or high-fat diet (23.43% Kcal of protein, 46.63% Kcal of carbohydrate, 30% Kcal of fat) according to the experimental group (Table 1). The main source of fat consisted of lard. After preparation, the feed was kept refrigerated until the time of consumption by the animals. In addition, the rats given high-fat diet groups also received water with 5% sucrose (high sugar) during the same period from day 90 to 120 of life and during pregnancy, which corresponds to reproductive age of adult rats.

**Table 1:** Nutritional values of diet offered to non-diabetic and diabetic rats.

**Information**	**Food (Kcal/g)**		**Water (Kcal/mL)**	
	**Commercial**	**High-fat**	**Standard**	**High-sugar**
Gross energy	4.02	4.93	–	0.20
Mixture (%)	7.45	3.65	100	100
Dry matter (%)	92.55	96.35	–	–
Mineral matter (%)	6.66	2.84	–	–
Crude protein (%)	25.76	26.77	–	–
Ether extract (%)	3.49	15.19	–	–
Gross fiber (%)	43.63	45.42	–	–
Carbohydrates (%)	13.01	6.13	–	5.00

### Mating

At 120 days of life, the female rats were similarly mated as their mothers. After 15 consecutive days, non-mated rats were considered infertile and excluded from the experiment. For this, three females were placed in the overnight period with normoglycemic males presenting similar age, which were purchased for this purpose. The next morning (7–9 a.m.) the male arts were removed and vaginal smears were performed in female rats. The presence of spermatozoa on the slides confirmed the diagnosis of pregnancy, which was considered zero pregnancy day (D0) (Damasceno et al., 2011).

### Course of Pregnancy—Diabetes and Obesity Features

Maternal body weight, food consumption, and water intake were measured every 7 days up to the end of pregnancy, at approximately 9 a.m. At days 0 and 17 of pregnancy, OGTT was again performed to evaluate glycemia. The glycemic values were used to mathematically estimate the total area under the curve (AUC) by the trapezoidal method (Tai, 1994; Gallego et al., 2019). For the obesity parameter, Lee Index was obtained at days 0 and 17 of pregnancy, and defined as the cube root of body weight (g) 10/nasoanal length (cm), for which a value equal to or <0.300 was classified as normal. Rats presenting values higher than 0.300 were classified as obese (Bernardis and Patterson, 1968; Soares et al., 2017).

At term pregnancy (day 21), the female rats were anesthetized with sodium thiopental (Thiopentax®, intraperitoneal route, 120 mg/kg according to protocols of Ethical Committee), and, after confirming the signs that showed successful anesthetic procedure, the animals were decapitated to obtain blood samples. Then, the rats were submitted to laparotomy for exposure of uterine horns. White adipose depots were collected around ovaries and then weighed.

### Biochemical and Hematological Profile Analysis

The blood samples were collected in dry tubes and maintained on ice for 30 min and then centrifuged at 1,575 × *g* for 10 min at 4°C. The serum supernatant was at −80°C for determination of triglycerides (TG), total cholesterol (TC), high-density lipoprotein cholesterol (HDL-c), using commercial kits.

For hematological analysis, blood was collected (500 μL) and transferred to tubes with anticoagulant (EDTA). The total leukocyte count was determined on blood samples diluted 1:20 in Turk's solution, using a Neubauer's hemocytometer. For differential white blood cell counting, blood smears were fixed with methanol and stained with Giemsa's solution. According to staining and morphological criteria, differential cell analysis was performed under the light microscope by counting 100 cells, and the percentage of each cell type was calculated.

### Reproductive Outcomes and Fetal Development

The gravid uterus was withdrawn and dissected for evaluation of live and dead fetuses, reabsorption (embryonic death), implantation, and corpora lutea numbers. The number of undetectable implantation sites was determined by the Salewski method (Salewski, 1964). The percentage of preimplantation loss was calculated by [(number of corpora lutea – number of implantation)/number of corpora lutea] ×100. The percentage of postimplantation loss was determined by [(number of implantation – number of live fetuses)/number of implantation] ×100 (Afiune et al., 2017). Following the collection of fetuses from the uterine horns, these were weighed and classified as small (SGA), adequate (AGA), or large (LGA) for gestational age (Moraes-Souza et al., 2017). The placentas were weighed to calculate the placental efficiency (fetal weight/placental weight) (Volpato et al., 2015).

After weight, each fetus was externally examined for cranial conformation, implantation of ears, eyes, and mouth (existence of a cleft lip), anterior and posterior limbs (absence or excess of fingers, position, and size of limbs), thoracic, abdominal, and dorsal regions (presence of hemorrhage, hematoma, and neural tube closure defect), tail (size and shape), and anal perforation. Half of the number of fetuses of each liter was fixed in Bodian's solution, and serial sections were prepared as described by Wilson (1965) for visceral examination. The other fetuses were processed for examination of the bones by the staining procedure of Staples and Schnell (1964). Besides the skeletal analyses, the counting of the ossification sites was performed according to methodology proposed by Aliverti et al. (1979), which determines the degree of fetal development. Fetuses that showed no external, skeletal, and visceral anomalies were considered normal.

### Statistical Analysis

The comparison of the mean values between the experimental groups was determined by analysis of variance (ANOVA), followed by Tukey's multiple comparison test. Student's *t*-test was used to compare difference of time (day 0 × day 21 of pregnancy). Proportions were calculated by the Fisher's exact test. To verify the normality of the results, the Shapiro–Wilk Normality test was used. Differences were considered statistically significant when *p* <0.05.

## Results

### Obesity Features

Table 2 shows obesity features. The rats NDHF/HS presented lower feed intake and higher water intake, a positive obesity rate at day 0 of pregnancy, periovarian, and visceral adipose tissue weight compared with the NDS group. The DS group showed decreased gravid uterus weight and higher periovarian/visceral adipose tissues weight when compared with NDS rats. The DHF/HS rats presented increase in water and caloric intake, number of obese rats, periovarian and visceral adipose tissue weight, decrease in feed intake, maternal weight gain, and gravid uterus weight compared with the NDS rats. In addition, the feed intake was increased, and water intake was decreased compared with NDHF/HS and DS groups; and the DHF/HS group had the gravid uterus weight decrease compared with the NDHF/HS rats and a higher positive obesity rate compared with the DS group.

**Table 2:** Obesity features of non-diabetic (ND) and diabetic (D) rats treated or not (S) with high-fat/high-sugar diet (HF/HS) before and during pregnancy.

	**Groups**			
	**NDS (*n* = 13)**	**NDHF/HS (*n* = 11)**	**DS (*n* = 12)**	**DHF/HS (*n* = 13)**
Food intake (g/day)^a^	19.7 ± 1.6	13.5 ± 1.5^*^	21.0 ± 2.4	16.4 ± 3.1^*^^#^^$^
Water intake (mL/day)^a^	44.2 ± 5.1	92.1 ± 23.3^*^	39.4 ± 13.9	72.0 ±18.8^*^^#^^$^
Caloric intake (Kcal/day)^a^	79.2 ± 6.4	84.9 ± 7.8	84.4 ± 9.6	95.2 ± 15.8^*^
Weight gain in pregnancy (g)^a^	116.7 ± 13.3	110.8 ± 16.6	90.7 ± 42.3	79.8 ± 39.9^*^
Gravid uterus weight (g)^a^	82.3 ± 13.2	82.9 ± 13.0	55.4 ± 31.4^*^	55.8 ± 26.9^*^^#^
**Positive obesity (%)^b^**
Previous pregnancy	0.0	71.4^*^	25.0^#^	77.8^*^^$^
Weight of periovarian adipose tissue (g)^a^	0.4 ± 0.1	0.9± 0.2^*^	0.8 ± 0.2^*^	1.1 ± 0.4^*^
Weight of visceral adipose tissue (g)^a^	3.1 ± 0.5	4.0 ± 1.5^*^	3.9 ± 0.9^*^	5.4 ± 1.8^*^

*Data shown as mean ± standard deviation (SD) and proportions (%)*.

* *p < 0.05, compared with the NDS group;*

# *p < 0.05, compared with the NDHF/HS group;*

$ *p < 0.05, compared with the DS group*

a *(ANOVA followed Tukey's multiple comparison test;*

b *Fisher's exact test)*.

### Diabetes Biomarker

**Figure 2:**
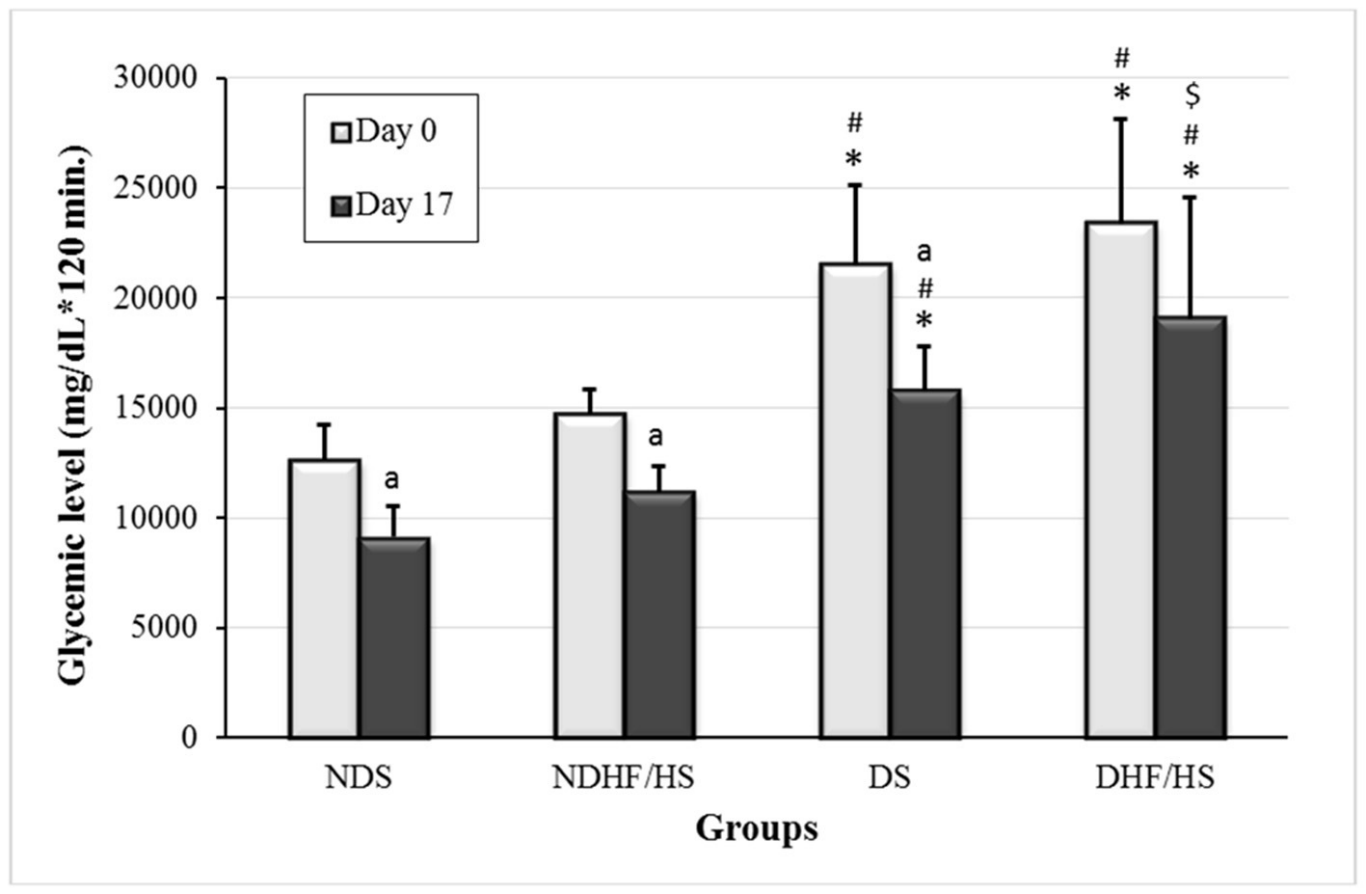
An area under the curve of oral glucose tolerance test (OGTT) on days 0 and 17 of pregnancy of non-diabetic (ND) and diabetic (D) rats treated or not (S), with high-fat/high-sugar diet (HF/HS) before and during pregnancy. Data shown as mean ± standard deviation (SD). *p <0.05, compared with the NDS group; ^#^*p* <0.05, compared with NDHF/HS group; ^$^*p* <0.05, compared with the DS group (ANOVA followed Tukey's Multiple Comparison test); ^a^*p* <0.05, compared with day 0 of pregnancy (Student's *T*-test).

The area under the curve (AUC) obtained by oral glucose tolerance test (OGTT) was increased in both diabetic groups (DS and DHF/HS) on days 0 and 17 of pregnancy compared with non-diabetic groups (NDS and NDHF/HS). In addition, the DHF/HS group showed an increase in AUC on day 17 of pregnancy compared with the DS group. There was no difference in AUC between days 0 and 17 of pregnancy of DHF/HS rats (Figure 2).

### Maternal Biochemical Parameters

There was no difference in TC, ALT, and AST concentrations among the groups. The DHF/HS group showed an increase in protein and albumin compared with the non-diabetic groups (NDS and NDHF/HS). The TG levels groups presented an increase in the NDHF/HS group compared with standard diet groups (NDS and DS), and the DHF/HS showed an increase in relation to other groups (Table 3).

**Table 3:** Biochemistry parameters at term pregnancy of non-diabetic (ND) and diabetic (D) rats treated or not (S) with high-fat/high-sugar diet (HF/HS) before and during pregnancy.

	**Groups**			
	**NDS (*n* = 13)**	**NDHF/HS (*n* = 11)**	**DS (*n* = 12)**	**DHF/HS (*n* = 13)**
Total protein (g/dL)	5.5 ± 0.9	5.5 ± 1.5	6.3 ± 1.1	8.1 ± 1.9^*^^#^
Albumin (mg dL)	2.8 ± 0.2	2.8 ± 0.4	3.1 ± 0.5	3.3 ± 0.4^*^^#^
TC (mg/dL)	61.4 ± 3.2	77.4 ± 12.4	66.0 ± 5.2	82.0 ± 9.8
TG (mg/dL)	97.2 ± 7.1	372.4 ± 119.9^*^	182.0 ± 34.3^#^	485.6 ± 138.5^*^^#^^$^
ALT (U/l)	87.2 ± 8.9	90.8 ± 6.3	84.0 ± 7.8	92.6 ± 10.8
AST (U/L)	188.4 ± 8.5	151.8 ± 21.6	181.8 ± 27.6	164.6 ± 30.9

*Data shown as mean ± standard deviation (SD)*.

* *p < 0.05, compared with the NDS group;*

# *p < 0.05, compared with the NDHF/HS group;*

$ *p < 0.05, compared with the DS group (ANOVA followed Tukey's multiple comparison test)*.

### Hematological Profile

The DS group showed decreased number of monocytes compared with the NDS rats. There was an increase in total leukocytes, segmented, and eosinophil in the DHF/HS group compared with the other experimental groups. The DHF/HS group also had increased number of monocytes in relation to the NDHF/HS and DS groups (Table 4).

**Table 4:** A hematological profile at term pregnancy of non-diabetic (ND) and diabetic (D) rats treated or not (S) with high-fat/high-sugar diet (HF/HS) before and during pregnancy.

	**Groups**			
	**NDS (*n* = 13)**	**NDHF/HS (*n* = 11)**	**DS (*n* = 12)**	**DHF/HS (*n* = 13)**
Leukocytes (103/mm3)	5.91 ± 0.82	5.47 ± 2.63	6.03 ± 0.82	8.64 ±1.65^*^^#^^$^
Segmented (103/mm3)	2.23 ± 0.31 (38–43%)	2.64 ± 1.60 (41–51%)	2.52 ± 0.43 (33–53%)	4.51 ± 1.24^*^^#^^$^ (44–60%)
Lymphocytes (103/mm3)	3.41 ± 0.73 (52–62%)	2.73 ± 1.01 (45–55%)	3.34 ± 1.02 (44–64%)	3.81 ± 1.01 (35–53%)
Monocytes (103/mm3)	0.20 ± 0.08 (2–4%)	0.12 ± 0.06 (1–3%)	0.09 ± 0.04^*^ (0–2%)	0.25 ± 0.08^#^^$^ (2–4%)
Eosinophils (103/mm3)	0.01 ± 0.02 (0–1%)	0.01 ± 0.01 (0–1%)	0.02 ± 0.03 (0–1%)	0.09 ± 0.08^*^^#^^$^ (0–2%)
Basophil (103/mm3)	0.00 ± 0.00 (0–0%)	0.00 ± 0.00 (0–0%)	0.00 ± 0.00 (0–0%)	0.00 ± 0.00 (0–0%)

*Data shown as mean ± standard deviation (SD)*.

* *p < 0.05, compared with the ND group;*

# *p < 0.05, compared with the NDHF/HS group;*

$ *p < 0.05, compared with the DS group (ANOVA followed Tukey's multiple comparison test)*.

### Pre- and Postimplantation Embryonic Losses

**Figure 3:**
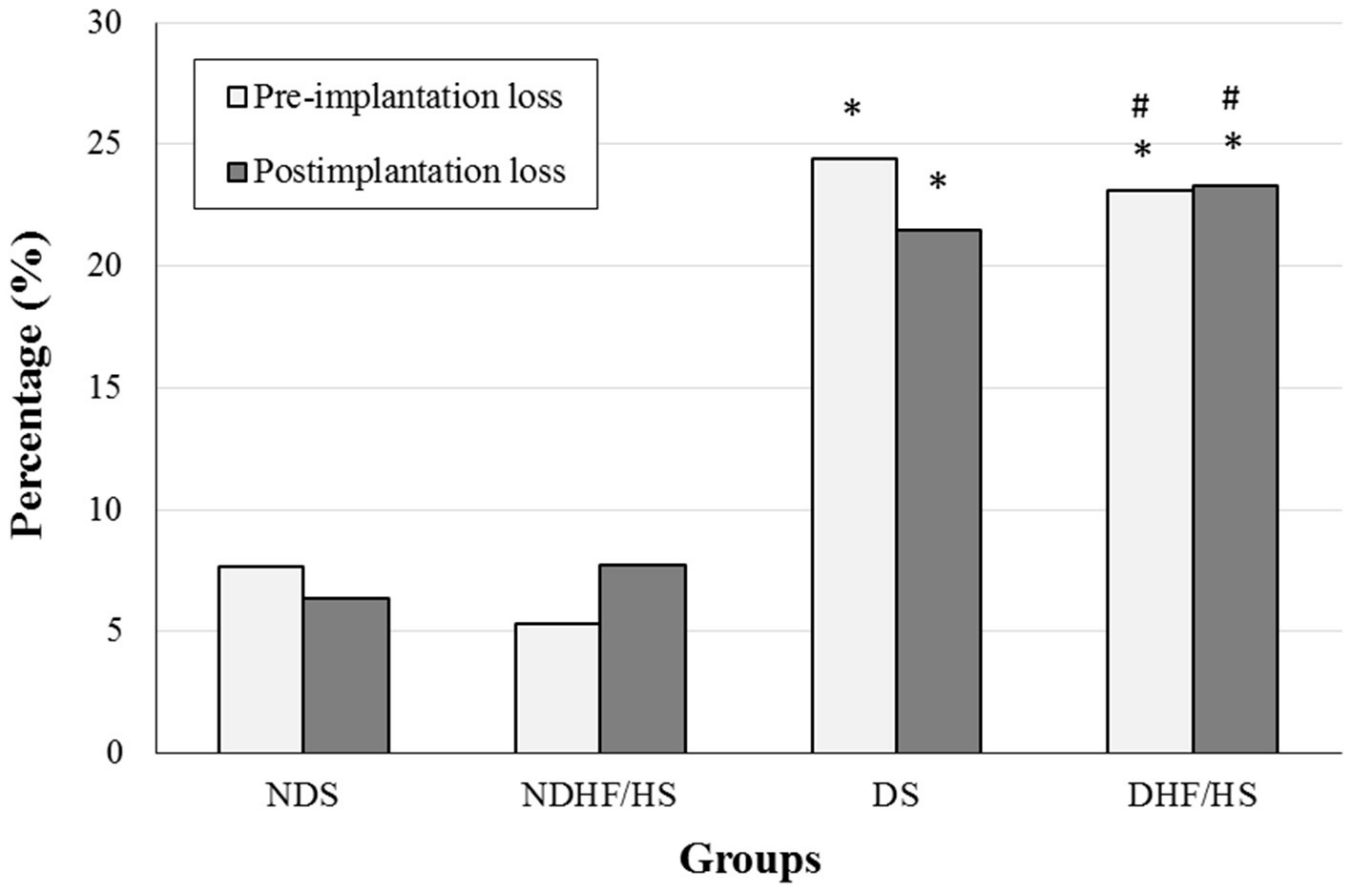
Percentage (%) of pre- and postimplantation losses of non-diabetic (ND) and diabetic (D) rats treated or not (S), with high-fat/high-sugar diet (HF/HS) before and during pregnancy. *p <0.05, compared with the NDS group; ^#^*p* <0.05, compared with the NDHF/HS group (Fisher's exact test).

Figure 3 shows the embryonic losses before and after the implantation process. The diabetic rats of both groups (DS and DHF/HS) showed an increased percentage of pre- and postimplantation losses compared with those of non-diabetic animals (NDS and NDHF/HS).

### Fetal and Placental Data

The fetal weight and number of fetuses classified as adequate for gestational age (AGA) were decreased in the NDHF/HS and DHF/HS groups compared with those of NDS group. The percentage of fetuses classified as small for gestational age (SGA) was increased in the NDHF/HS and DHF/HS rats in relation with those of the NDS group. The NDHF/HS group presented decrease in placental weight compared with the other groups, and the DHF/HS rats showed decrease in placental efficiency compared with the NDS rats. The ossification sites of fetuses from dams that received HF/HS diet (ND and D) were decreased in relation to respective control groups (Table 5).

**Table 5:** Fetal and placental weights, placental efficiency, and ossification sites of fetuses from non-diabetic (ND) and diabetic (D) rats treated or not (S) with high-fat/high-sugar diet (HF/HS) before and during pregnancy.

	**Groups**			
	**NDS (*n* = 147 fetuses)**	**NDHF/HS (*n* = 131 fetuses)**	**DS (*n* = 95 fetuses)**	**DHF/HS (*n* = 102 fetuses)**
Fetal weight (g)^a^	5.57 ± 0.46	5.25 ± 0.65^*^	5.46 ± 0.49	5.35 ± 0.55^*^
SGA Fetuses (%)^b^	4.08	19.08^*^	10.54	16.66^*^
AGA Fetuses (%)^b^	90.48	75.58^*^	84.20	80.40^*^
LGA Fetuses (%)^b^	5.44	5.34	3.16	2.94
Placental weight (g)^a^	0.49 ± 0.06	0.47 ± 0.07^*^	0.49 ± 0.08^#^	0.50 ± 0.09^#^
Placental efficiency^a^	11.58 ± 1.42	11.45 ± 1.85	11.24 ± 1.68	10.82 ± 1.73^*^
Ossification sites^a^	24.96 ± 1.71	22.80 ± 2.02^*^	24.42 ± 2.49	21.78 ± 0.93^*^^$^

*Data shown as mean ± standard deviation (SD) and proportions (%)*.

* *p < 0.05, compared with the C Group;*

# *p < 0.05, compared with the NDHF/HS group;*

$ *p < 0.05, compared with the D Group (^a^ANOVA followed Tukey's multiple comparison test; ^b^Fisher's exact test)*.

### Fetal Anomalies

**Figure 4:**
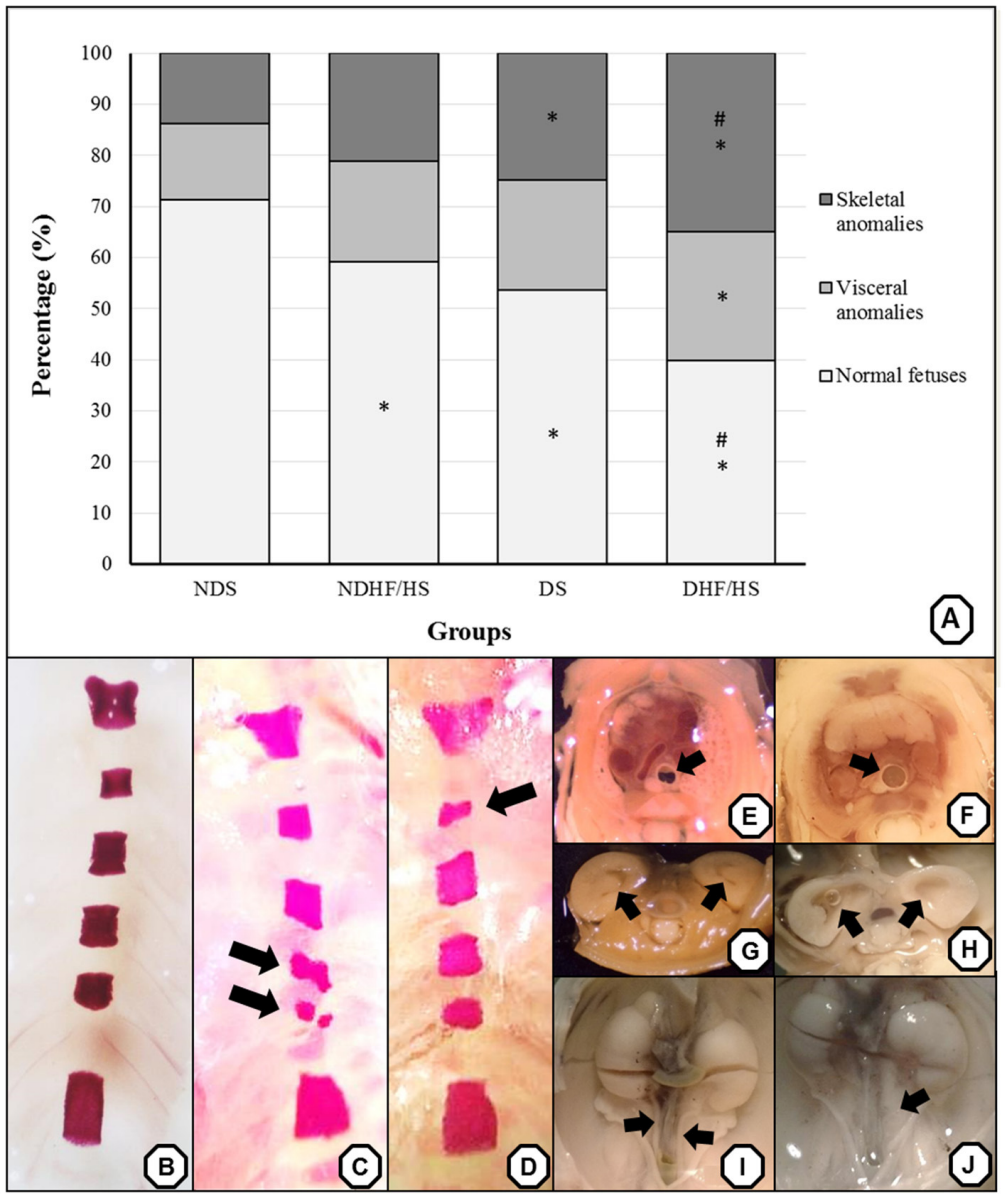
**(A)** Percentage (%) of anomalies of fetuses from non-diabetic (ND) and diabetic (D) rats in treated or not (S) with high-fat/high-sugar diet (HF/HS) before and during pregnancy. Panels **(B–J)**—representative images of the main skeletal **(B–D)** and visceral **(F–J)** anomalies. Panel **(B)**—normal sternebra of rat fetuses. Panel **(C)**—abnormally shaped sternebra and bipartite ossification of sternebra (arrow). Panel **(D)**—incomplete ossification of sternebra (arrow). Panels **(E,F)**—a thoracic section from rat fetuses, with **(E)** normal trachea (arrow) and **(F)** dilated trachea (arrow). Panels **(G,H)**—a kidney transversal section from rat fetuses, with **(G)** normal renal calices (arrow) and **(H)** dilated renal calices (arrow). Panels **(I,J)**—the pelvis section from rat fetuses, with **(I)** normal ureter (arrow) and **(J)** enlarged ureter—hydroureter (arrow). *p <0.05—compared with the NDS group; ^#^*p* <0.05, compared with the NDHF/HS group (Fisher's exact test).

The percentage of normal fetuses was decreased in three groups when compared with the ND group. The diabetic groups (DS and DHF/HS) showed an increased percentage of fetuses with skeletal anomalies compared with the ND groups. The fetuses of the DHF/HS rats showed higher incidence of visceral anomalies compared with those of the ND group, higher percentage of skeletal anomalies and lower percentage of normal fetuses compared with those of the NDHF/HS group (Figure 4A). Representative images of the main anomalies found are shown in Figures 4B–J.

## Discussion

The streptozotocin-induced mild diabetes in rat offspring after birth caused a diabetic status. This was confirmed by oral glucose tolerance test (OGTT) and higher area under curve (AUC) data. Before pregnancy, there was no significant difference in fertility rates among the groups. In the experimental model of mild diabetes induction, used in the present study, the fertility rate was around 90%, corroborating Sinzato et al. (2021). In the groups that consumed HS/HF diet, the treatment time was not enough to change the fertility rate. During pregnancy of these rats, the hyperglycemia led to impairment on embryonic development, contributing to embryo losses as verified at term pregnancy. The rats that received high-fat diet and sugar in drinking water at adulthood presented with greater Lee index values, confirming obesity. In addition, these female rats showed higher periovarian and visceral fat weight and hypertriglyceridemia. In this maternal condition, there was higher incidence of small fetuses for gestational age, indicating intrauterine growth restriction (IUGR) associated with obesity. The use of animal models to study maternal association between diabetes and obesity helps understand the functional, biochemical, and morphological changes caused by these connected diseases. Our findings showed that diabetes and obesity status caused maternal hyperglycemia and an abnormal leukocyte profile, contributing to IUGR. In addition, this association led to embryo-fetal losses and the onset of anomalies in the fetuses at the end of pregnancy, confirming the maternal, fetal, and perinatal complications induced by diabetes and obesity during pregnancy.

Metabolic disorders, such as diabetes, may cause hyperphagia condition, but the mechanisms involved are not fully understood (Li et al., 2019). The regulator of food intake is influenced by the balance among appetite, satiety, and energy expenditure, and this biological process is called “energy homeostasis” (Morton et al., 2014; Deemer et al., 2019). This balance is regulated by the central nervous system (Deemer et al., 2019) and multiple metabolic signals, such as leptin (Zhang and Chua, 2017), insulin (Brüning et al., 2000), glucagon-like peptide 1 (GLP1) (Ong et al., 2017), and cholecystokinin (CCK) (Woods et al., 2018). In this study, diabetes and high-fat/high-sugar diet alone did not interfere with daily caloric intake. However, the association between diabetes and abnormal diet might activate neurocircuits, which impaired the controller system of energy homeostasis, influencing in body weight (Morton et al., 2014) and the regulation of caloric intake. Then, even the rats eating less high-fat diet but drinking more sugar in the water presented obesity status. The deregulation of energy consumption is one of the major causes of obesity (Erlanson-Albertsson, 2005), confirmed by body weight (de Almeida et al., 2016), body composition and fat deposits, especially visceral fat (Poirier et al., 2006). Our findings showed that diabetic rats submitted to a high-fat/high-sugar diet showed a reduction in body weight gain during pregnancy, but, even so, they developed obesity, as verified by Lee index, which is a murinometric parameter for obesity classification used in experimental studies (Bernardis and Patterson, 1968; Fernandes et al., 2012). Concomitantly, there was an increased weight of periovarian and visceral fat. In experimental models, the carcass relative fat is one of the variables to indicate obesity (Nascimento et al., 2008; Kim et al., 2017).

The oral glucose tolerance test (OGTT) determines degree of glucose tolerance, expressing the ability of β-pancreatic cells to secrete insulin and tissue sensitivity to this hormone (American Diabetes Association, 2020). There were higher blood glucose values in the OGTT in diabetic groups and consequent increase in the area under the curve (AUC), leading to glucose intolerance and, later, diabetes. Then, once diabetes and obesity have been confirmed, it was demonstrated that diabetic and obese rats presented higher levels of blood total protein and albumin. These biochemical parameters are used in animal nutrition research to evaluate its health (Luca and Reis, 2004). Several processes regulate plasma albumin concentration, including synthesis, distribution, and exogenous albumin loss (Dom and Kaysen, 2003). Roche et al. (2008) and Guerin-Dubourg et al. (2012) described albumin as an antioxidant, and it might be elevated in our animals to compensate the higher levels of reactive oxygen species (ROS) induced by diabetes (Raza et al., 2011; Patche et al., 2017; Sinzato et al., 2019) and obesity (Diniz et al., 2004; Burneiko et al., 2006; De Sibio et al., 2013). Our results showed that obesity, alone or associated with diabetes, causes dyslipidemia. Other authors also verified dyslipidemia in experimental animals (Panchal et al., 2011; Zhou et al., 2014; Hao et al., 2015; Senaphan et al., 2015), and different types of diets influence the lipid profile (Desroches et al., 2006). Lipid metabolism, including lipid absorption, transport, synthesis, and degradation, is a complex process, which can lead to other diseases (Huang and Freter, 2015). Among these, diabetes (Dong et al., 2017), inflammation, atherosclerosis (Joseph et al., 2003), obesity (Kaess et al., 2014), and hypertension (Siri-Tarino and Krauss, 2016) are related. The male rats feeding HFD showed increased blood pressure (Sá et al., 2019), and Hsu et al. (2019) showed that the consumption of HFD during pregnancy of rats was responsible for inducing hypertension in adult offspring.

Considering the hematological profile, the association between diabetes and obesity increased the number of total leukocytes, segmented (mature neutrophils), monocytes, and eosinophil. The total and differential leukocyte count is an important parameter to evaluate conditions related with inflammatory processes (George-Gay and Parker, 2003). Increased leukocyte amount due to deregulation of immune activity caused by adipose tissue expansion contributes to obesity-induced inflammation (Trellakis et al., 2012; Poret et al., 2018). Obesity may cause immunomodulation, inducing a higher ratio from neutrophils to lymphocytes due to increased recruitment and activation of peripheral blood neutrophils to adipose tissue (Elgazar-Carmon et al., 2008; Trellakis et al., 2012). In addition, it can stimulate mobilization of bone marrow monocytes so that they fall into the bloodstream and reach adipose tissue as macrophages (Ghigliotti et al., 2014). In this study, the diabetic rats presented lower levels of monocytes. Monocyte is one of the main leukocyte subtypes and is considered an inflammatory biomarker (Badr et al., 2019), and its influx in perivascular regions and retinal pigment epithelium has been verified (Benhar et al., 2016). Decreased peripheral blood monocyte levels were related to diabetic retinopathy in diabetic adults without potential confounders (Wan et al., 2020), suggesting the onset of the diabetes-induced retinal complication in these dams. Eosinophils are the main regulators of the physiological processes and immune function of perivascular adipose tissue (Withers et al., 2017). According to Maizels and Allen (2011), eosinophils prevent inflammation caused by obesity because it possibly increases the numbers of eosinophils or Th2 cells. This might be explained because the IL-4 and IL-13 secretion signal gamma peroxisome proliferator activated receptor (PPARγ), which, if activated by appropriate lipids, inhibits the expression of genes that promote inflammation (Szanto et al., 2010). Therefore, it is supposed that eosinophilia present in the diabetic and obese group was due to the homeostatic mechanism, tending to minimize the possible inflammation caused by obesity.

The reproductive analysis of the animals in this study showed embryonic losses before and after implantation, which were higher in both diabetic groups, demonstrating the influence of hyperglycemia on the implantation process. Regardless of the degree of severity, hyperglycemia is related to pre- and postimplantation losses in the intrauterine environment (Sinzato et al., 2011; Bequer et al., 2018; Gallego et al., 2018). Moreover, problems with cytokine regulation, which occurs in diabetic pregnancy, can lead to damage during early embryonic development, such as pre-implant failure, leading to a reduced number of implants and postimplantation losses indicated by an increased rate of resorption and a reduced number of live fetuses (Sinzato et al., 2011; Dela Justina et al., 2017). These findings contributed to a lesser weight gain during pregnancy and maternal final weight at term pregnancy. However, obesity did not increase embryo loss rates in the animals.

For the success of pregnancy, it is essential that, during the implantation period, the physiological and molecular processes are coordinated, involving close interactions between the uterus and the blastocyst (Cha et al., 2012). Then, the dams presenting biochemical alterations induced by diabetes, obesity, and both contributed to impaired reproductive outcomes. In relation to fetal development and growth from diabetic and/or obese dams, our study demonstrated that only altered diet caused intrauterine growth restriction, which was confirmed by reduction of the fetal weight, higher percentage of small fetuses for the gestational age, and decline of ossification sites. These findings corroborate other authors since diabetes (Damasceno et al., 2014) and maternal obesity and high-calorie intake (Zou et al., 2017) may impair fetal development. Growth restriction may be related to different maternal adaptations to diet and diet components, with maternal nutrition being a possible factor in intrauterine growth restriction (Howie et al., 2009; Setia and Sridhar, 2009; Mark et al., 2011; Tellechea et al., 2017). The animals that received a high fat/sugar diet had altered placental weights, which may be related to functional or morphological placental alterations. These placental changes may have contributed to the decrease in fetal weight. Intrauterine growth restriction leads to a variety of phenotypes related to the metabolic syndrome in adult children, including hypertension (Tain et al., 2017; Bendix et al., 2020). In addition, the obesity induced by high-fat/high-sugar diet, whether associated or not associated with diabetes, decreased the frequency of fetuses without anomalies. The diabetes status increased skeletal anomalies. The rats presenting diabetes and obesity showed an exacerbated percentage of fetal abnormalities, with an increased frequency of visceral anomalies. Bueno et al. (2020) already demonstrated that maternal hyperglycemia causes an abnormal fetal metabolism, contributing to an increase of visceral anomalies in the offspring of diabetic rats. Besides, fetal metabolic dysregulation may also occur due to the maternal consumption of a carbohydrate and lipid-rich diet (Musial et al., 2017), which interferes with various pathways of the developing organs, such as the liver, skeletal muscle, adipose tissue, brain, and pancreas (Heerwagen et al., 2010).

This study points out strengths as several variables and biomarkers evaluated, using a solid and structured experimental model; however, it is a study performed in laboratory animals and needs more attention for human application. For limitation, we have the lack of measurement of leptin, since it could be used during the discussion of appetite and feed intake. Another limiting factor is the determination of the free fat acid levels to relate to other lipid parameters.

In conclusion, the association between maternal diabetes and obesity induces metabolic, leukocyte, and biochemical alterations that contribute to affect fetal development and growth. Further studies are needed to clear more the mechanisms involved during diabesity in pregnancy to prevent the fetal/neonatal outcomes in humans. Then, the experimental model employed in our study helps understand some pathophysiological mechanisms linked to this association, allowing interventionist methods to avoid maternal changes and, consequently, fetal repercussions as found in this study.

## Data Availability Statement

The original contributions presented in the study are included in the article/supplementary material, further inquiries can be directed to the corresponding author/s.

## Ethics Statement

The animal study was reviewed and approved by The Ethical Committee for Animal Research of Araguaia—UFMT, Brazil (Protocol number 23108.022251/2019-61).

## Author Contributions

VA-S and GV: conception and design, data acquisition, data analysis and interpretation, and manuscript writing. AS-S, AL, and CB-B: data acquisition, data analysis and interpretation, and manuscript writing. RM-S, TS, VP, and YS: data interpretation and manuscript writing. BK: manuscript writing. DD: data analysis and interpretation and manuscript writing. All authors have reviewed the manuscript, agreed with its contents, consented to its publication, and that there are no other persons who satisfied the criteria for authorship. We further confirm that all of us have approved the order of the authors listed in the manuscript.

## Conflict of Interest

The authors declare that the research was conducted in the absence of any commercial or financial relationships that could be construed as a potential conflict of interest.

## Publisher's Note

All claims expressed in this article are solely those of the authors and do not necessarily represent those of their affiliated organizations, or those of the publisher, the editors and the reviewers. Any product that may be evaluated in this article, or claim that may be made by its manufacturer, is not guaranteed or endorsed by the publisher.
